# CRISPR/Cas‐based screening of a gene activation library in *Saccharomyces cerevisiae* identifies a crucial role of *OLE1* in thermotolerance

**DOI:** 10.1111/1751-7915.13333

**Published:** 2018-11-05

**Authors:** Pengsong Li, Xiaofen Fu, Lei Zhang, Shizhong Li

**Affiliations:** ^1^ MOST‐USDA Joint Research Center for Biofuels Beijing Engineering Research Center for Biofuels Institute of New Energy Technology Tsinghua University Beijing 100084 China

## Abstract

CRISPR/Cas‐based (clustered regularly interspaced short palindromic repeats/CRISPR‐associated) screening has been proved to be an efficient method to study functional genomics from yeast to human. In this study, we report the development of a focused CRISPR/Cas‐based gene activation library in *Saccharomyces cerevisiae* and its application in gene identification based on functional screening towards improved thermotolerance. The gene activation library was subjected to screening at 42°C, and the same library cultured at 30°C was set as a control group. After five successive subcultures, five clones were randomly picked from the libraries cultured at 30 and 42°C, respectively. The five clones selected at 30°C contain the specificity sequences of five different single guide RNAs, whereas all the five clones selected at 42°C contain the specificity sequence of one sgRNA that targets the promoter region of *OLE1*. A crucial role of *OLE1* in thermotolerance was identified: the overexpression of *OLE1* increased fatty acid unsaturation, and thereby helped counter lipid peroxidation caused by heat stress, rendering the yeast thermotolerant. This study described the application of CRISPR/Cas‐based gene activation screening with an example of thermotolerant yeast screening, demonstrating that this method can be used to identify functional genes in yeast.

## Introduction

Biofuels such as bioethanol are becoming increasingly important due to their ability to reduce fossil fuel consumption and reduce greenhouse gas and pollutant emissions (Goldemberg, 2007; Salvo *et al*., 2017). Large‐scale biofuel production benefits greatly from high‐temperature fermentation (≥ 40°C) which significantly reduces cooling costs and helps prevent contamination (Abdel‐Banat *et al*., 2010; Li *et al*., 2017). High operating temperature also benefits a simultaneous saccharification and fermentation (SSF) process because the optimal temperature for enzymes that catalyse the saccharification of biomass is usually over 50°C (Li *et al*., 2013; Caspeta *et al*., 2014a,b). However, industrial biofuel production usually employs mesophilic yeasts whose optimal growth temperatures range from 25 to 37°C. Among them, *Saccharomyces cerevisiae* is the most widely used industrial yeast species (Steensels *et al*., 2014). High temperature seriously destroys cytoskeletal integrity, causes cell morphological abnormalities, inhibits cell division and growth, and impacts metabolic activity (Torija *et al*., 2003; Guyot *et al*., 2005). Great efforts have been made in order to understand the mechanism of yeast thermotolerance and improve it (Caspeta *et al*., 2014a,b, 2016; Shui *et al*., 2015; Jia *et al*., 2016; Li *et al*., 2017). Nevertheless, yeast thermotolerance is a complex phenotype, involving synergistic actions of many genes and thereby being difficult to engineer (Santos and Stephanopoulos, 2008). In recent years, CRISPR/Cas‐based (clustered regularly interspaced short palindromic repeats/CRISPR‐associated) screening has been proved to be an efficient method to study functional genomics from yeast to human (Zhou *et al*., 2014; Smith *et al*., 2016; Joung *et al*., 2017), developing a new perspective for research on complex phenotypes. The naturally occurring CRISPR system requires two noncoding CRISPR RNAs (crRNAs) [including a trans‐activating crRNA (tracrRNA) and a precursor crRNA (pre‐crRNA)] and an endonuclease Cas9 which can be directed by the crRNAs via base pairing to the target genomic loci followed by a protospacer‐adjacent motif (PAM) to achieve double‐stranded DNA breaks (DSBs; Jinek *et al*., 2012). In the synthetically reconstituted system, these two short RNAs are fused into a single guide RNA (sgRNA). A Cas9 mutant with undetectable endonuclease activity (dCas9) fused with an effector (e.g., activator and repressor) domain has been used in eukaryotes for efficient gene activation or repression (Farzadfard *et al*., 2013; Gilbert *et al*., 2013, 2014; Perez‐Pinera *et al*., 2013; Joung *et al*., 2017). Taking advantage of the dCas9‐based gene regulation system, we developed a focused CRISPR/Cas‐based gene activation library that enables gene identification from high‐temperature screening in *S. cerevisiae*. The crucial role of *OLE1* in thermotolerance was identified.

## Results and discussion

### Construction and high‐temperature screening of the gene activation library

**Figure 1:**
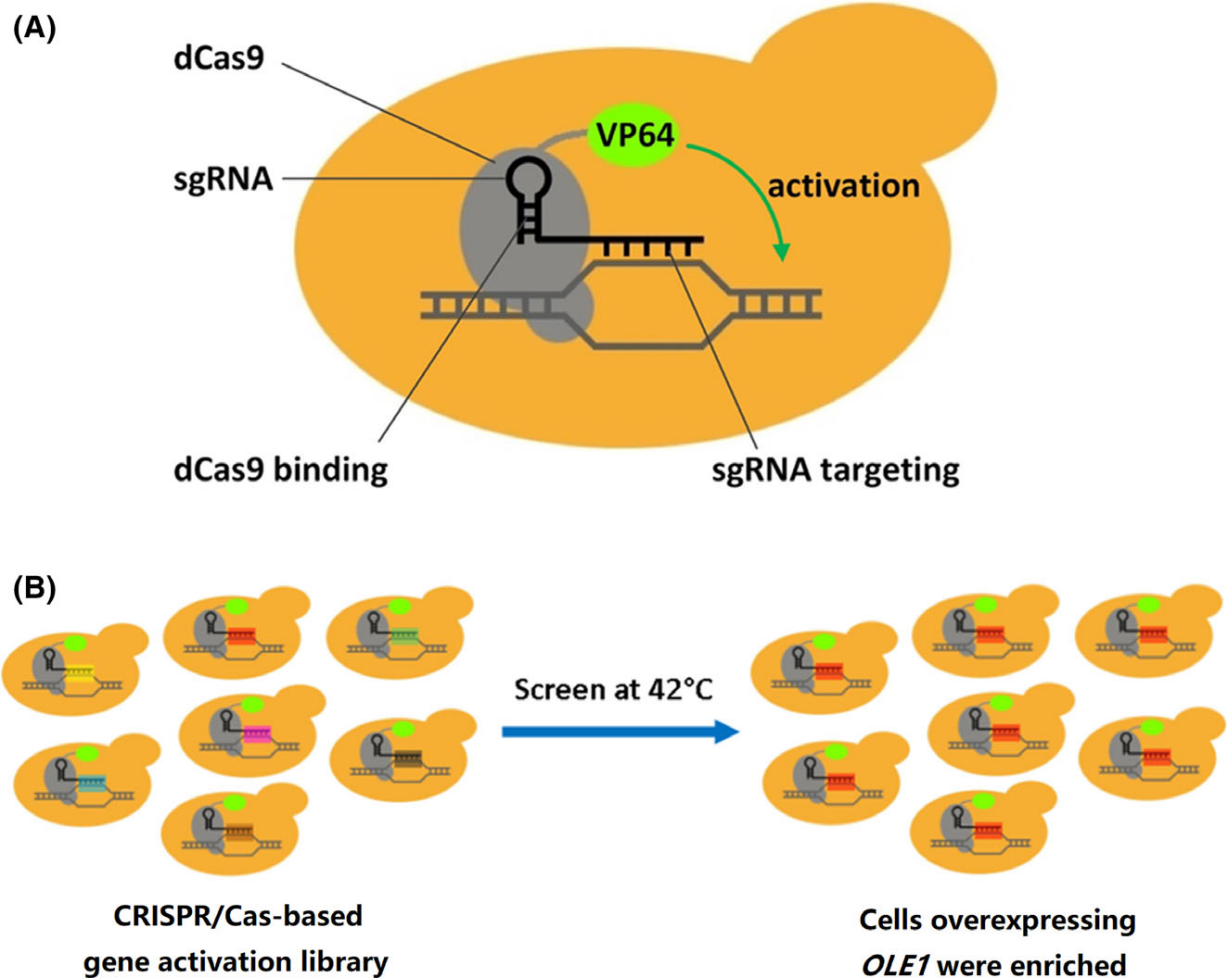
Schematic of (A) CRISPR activation (CRISPRa) system and (B) CRISPR/Cas‐based screening of the gene activation library in this study.

In our previous study, we have found that heterologous expression of *K. marxianus HSF1* and *MSN2* (denoted as *KmHSF1* and *KmMSN2*, respectively) in *S. cerevisiae* promoted cell growth and ethanol fermentation at high temperatures (Li *et al*., 2017). RNA‐Seq‐based transcriptomic analysis revealed that heterologous expression of *KmHSF1* and *KmMSN2* in *S. cerevisiae* resulted in 31 and 32 up‐regulated genes, respectively (Fold change > 2, *P*_adj_ < 0.05). Given that overexpression screening assay in yeast has been proved to be a powerful tool to identify functional genes or pathways that confer resistance to environmental stresses (Butcher *et al*., 2006; Jones *et al*., 2008), we employed CRISPR/Cas‐based gene activation screening to find the key functional genes that are involved in thermotolerance and further investigated the mechanism of yeast thermotolerance. The plasmid pScCRPa was constructed in order to activate the expression of target genes. The SV40 nuclear localization sequence (NLS) and four tandem copies of Herpes Simplex Viral Protein 16 (VP64, a commonly used eukaryotic transcription activator domain) were fused to a dCas9. The dCas9‐VP64 fusion is constitutively expressed from the *ADH1* promoter and guided to the target sites by the sgRNAs, which are constitutively expressed from the *SNR52* promoter and bind to the respective target sites (Fig. 1A and Fig. S1).

Thanks to the results of previous comparative transcriptomic analysis (Li *et al*., 2017), we could narrow down the library size towards more focused functional screens. We created a focused sgRNA library targeting the promoter regions of the up‐regulated genes (52 in total) found in our previous study (Li *et al*., 2017) and used it to generate a gene activation library of *S. cerevisiae* which was subsequently grown at 42°C for high‐temperature screening (Fig. 1B). Detailed information of the all the sgRNAs and paired oligonucleotides is listed in Table S1. After transformation into *E. coli* TOP10, ~3000 colonies were collected and pooled together for plasmid extraction, enabling the desired probability that any sgRNA occurs at least once in the library to be > 99.99% (Clarke and Carbon, 1976). Pooled plasmids containing the sgRNA cassettes were then transformed into *S. cerevisiae* TSH3 using *S. c*. EasyComp transformation kit (Life Technologies, Carlsbad, CA, USA) to generate the *S. cerevisiae* gene activation library.

The gene activation library was then subjected to screening at 42°C, and the same library cultured at 30°C was set as a control group. After five successive subcultures, five clones were randomly picked from the library cultured at 42 and 30°C, respectively. Surprisingly, all the five clones from the library cultured at 42°C contain one sgRNA specificity sequence (g*OLE1*_1 in Table S1) that targets the promoter region of *OLE1*, whereas the five clones from the control group contains five different sgRNA specificity sequences (g*GPH1*_4, g*FAS2*_4, g*HXT6*_3, g*PGI1*_1 and g*ANB1*_4 in Table S1) targeting the promoter regions of *GPH1*,* FAS2*,* HXT6*,* PGI1* and *ANB1*, respectively. Suppose that the 260 sgRNAs were evenly distributed in the library containing 3120 clones (just for convenience of calculation) after screening, every 12 clones contain one same sgRNA specificity sequence. When five clones were randomly picked from the library, the probability that these five clones contains five different sgRNAs specificity sequences (*P*_diff_), i.e. the probability of the above results of the control library screened at 30°C, can be calculated as follows: *P*_diff_ = _260_ *C*_5_ × (_12_ *C*_1_)^5^/_3120_ *C*_5_ = 0.965; and the probability that these five clones contain one same sgRNA specificity sequence (*P*_same_), i.e. the probability of the above results of screening at 42°C, can be calculated as follows: *P*_same_ = _260_ *C*_1_ × _12_ *C*_5_/_3120_ *C*_5_ = 8.38 × 10^−11^, which is thought to be statistically impossible. That is to say that it was statistically impossible to randomly pick five clones that contain one same sgRNA specificity sequence if the 260 sgRNAs were still evenly distributed in the library after high‐temperature screening. Therefore, we can deduce that yeast cells overexpressing *OLE1* were significantly enriched after high‐temperature screening (Fig. 1B).

**Figure 2:**
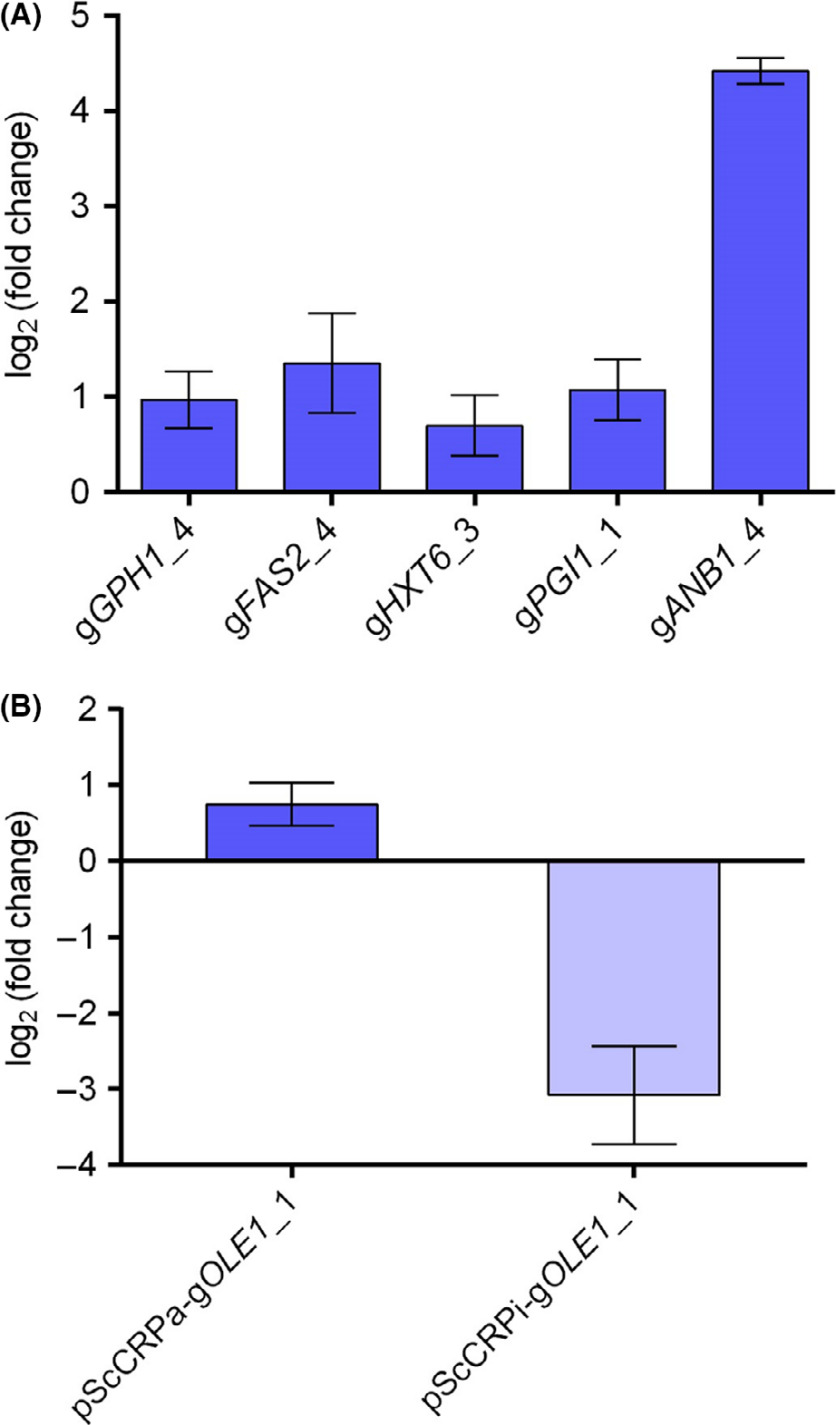
qRT‐PCR results (*TAF10* was used as internal reference gene).A. Fold changes of relative expression levels of *GPH1*,*FAS2*,*HXT6*,*PGI1* and *ANB1* in the five randomly picked strains compared to their relative expression levels in the strain harbouring blank plasmid pScCRPa.B. Fold changes of *OLE1* expression in strains harbouring pScCRPa‐g*OLE* *1*_1 and pScCRPi‐g*OLE* *1*_1 compared to its relative expression level in the strain harbouring blank plasmid pScCRPa.

Then, we conducted real‐time quantitative reverse transcription PCR (qRT‐PCR) to examine whether the CRISPR activation (CRISPRa) system in this study can up‐regulate expression of the five genes selected at 30°C. As shown in Fig. 2A, *GPH1*,* FAS2*,* HXT6*,* PGI1* and *ANB1* were found up‐regulated by 1.96‐, 2.56‐, 1.62‐, 2.10‐ and 21.37‐fold in corresponding strains, respectively. This indicates that the CRISPRa system in this study was effective. However, there is a great difference in fold change of gene expression, suggesting that activation efficiency of sgRNA varies from each other. Smith *et al*. (2016) reported that the best region to target sgRNA is between the transcription start site (TSS) and 200 bp upstream of the TSS in *S. cerevisiae*. As shown in Table S1, the targeting sites of all the selected sgRNAs except g*HXT6*_3 were in this optimal region. This can explain why the fold change of *HXT6* up‐regulation was the lowest among the five selected genes. Surprisingly, the fold change of *ANB1* up‐regulation was about 10 times those of other selected genes, indicating that g*ANB1*_4 was much more efficient in gene activation than other selected sgRNAs. In fact, the results of CRISPR‐dCas9‐based gene regulation often vary by orders of magnitude between loci and between different sgRNAs at the same locus (Jusiak *et al*., 2016). Therefore, multiple sgRNAs need to be designed for each gene in order to avoid unsuccessful regulation caused by inefficient sgRNAs.

### The overexpression of OLE1 enhanced thermotolerance

In order to examine whether the overexpression of *OLE1* confers thermotolerance to the yeast, we re‐constructed a CRISPRa‐based plasmid for *OLE1* overexpression, pScCRPa‐g*OLE1*_1, and re‐transformed it into TSH3. This strain contains the specificity sequence of g*OLE1*_1 that was enriched through high‐temperature screening. Two strains were also constructed as blank control and negative control, respectively. The blank control harbours the pScCRPa plasmid without any specificity sequences of sgRNAs, while the negative control harbours the plasmid pScCRPi‐g*OLE1*_1 (Fig. S2). The plasmid pScCRPi‐g*OLE1*_1 contains the specificity sequence of g*OLE1*_1 and a dCas9 fused to an NLS and an Mxi1 domain (Farzadfard *et al*., 2013), which is used to repress the expression of *OLE1*. According to the results of qRT‐PCR, *OLE1* was found up‐regulated by 1.65‐fold in the strain harbouring pScCRPa‐g*OLE1*_1 and down‐regulated by 7.39‐fold in the strain harbouring pScCRPi‐g*OLE1*_1, when compared to that in the blank control strain (Fig. 2B). These results indicate that under the guidance of g*OLE1*_1, the expression of *OLE1* was activated by the dCas9‐VP64 fusion and repressed by the dCas9‐Mxi1 fusion in corresponding strains as expected. Even though the fold change of *OLE1* up‐regulation was relatively low, *OLE1*‐overexpressing strain could be enriched after high‐temperature screening, indicating that this level of up‐regulation was sufficient to cause phenotype change. Given that *OLE1* was up‐regulated by 1.65‐fold in the strain overexpressing *KmMSN2* based on the RNA‐Seq results in our previous study (Li *et al*., 2017), the activation of *OLE1* based on the dCas9‐VP64 fusion and g*OLE1*_1 in this study is comparable to that by *KmMSN2*.

**Figure 3:**
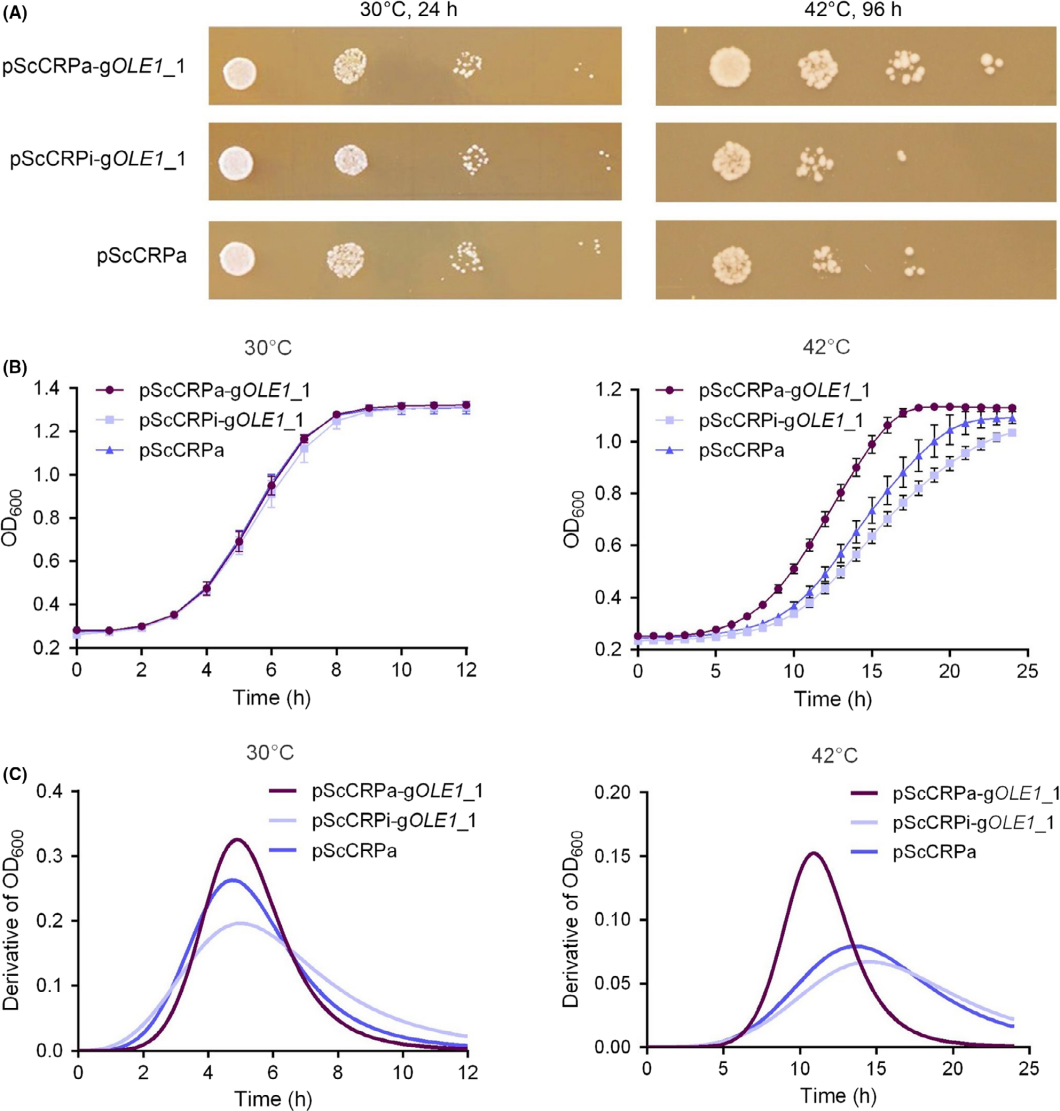
Effects of *OLE1* on cell growth at high temperature (42°C).A. Spotting tests showing the viability of different strains.B. Growth curves of different strains. Logistic model was used for data fitting.C. The first‐order derivative of the logistic functions in Fig. 3B showing the growth rate changes of different strains. pScCRPa‐g*OLE* *1*_1, *OLE1* overexpression strain; pScCRPi‐g*OLE* *1*_1, *OLE1* knockdown strain as negative control; pScCRPa, strain harbouring blank plasmid pScCRPa as blank control.

Then we conducted spotting tests and growth curve assays to investigate the effect of *OLE1* expression on cell growth at 30 and 42°C. As shown in Fig. 3A, the strains exhibited no obvious difference in viability between each other at 30°C. At 42°C, however, the *OLE1*‐overexpressing strain exhibited higher viability than the control stains (Fig. 3A). Moreover, the *OLE1*‐overexpressing strain grown faster than the control strains at 42°C, but no significant difference between growth curves of different strains was observed at 30°C (Fig. 3B). The growth curve data were fitted with logistic model, and the results show that all sets of data fitted the model well (*R* ^2^ > 0.99; Table S2). Then, the first‐order derivative functions of growth curves were calculated to show the variations of growth speed as a function of time. At 30°C, although the maximum growth speed of the *OLE1*‐overexpressing strain was greater than the control strains, the maximum growth speed of all the three strains appeared almost simultaneously at ~5 h after inoculation (Fig. 3C). At 42°C, the maximum growth speed of the *OLE1*‐overexpressing strain appeared at ~11 h inoculation, but those of the blank control and negative strains happened ~2.5 and ~3 h later, respectively (Fig. 3C). All the above results indicate that the overexpression of *OLE1* enhanced yeast thermotolerance.

### OLE1 enhanced thermotolerance by reducing lipid peroxidation

**Figure 4:**
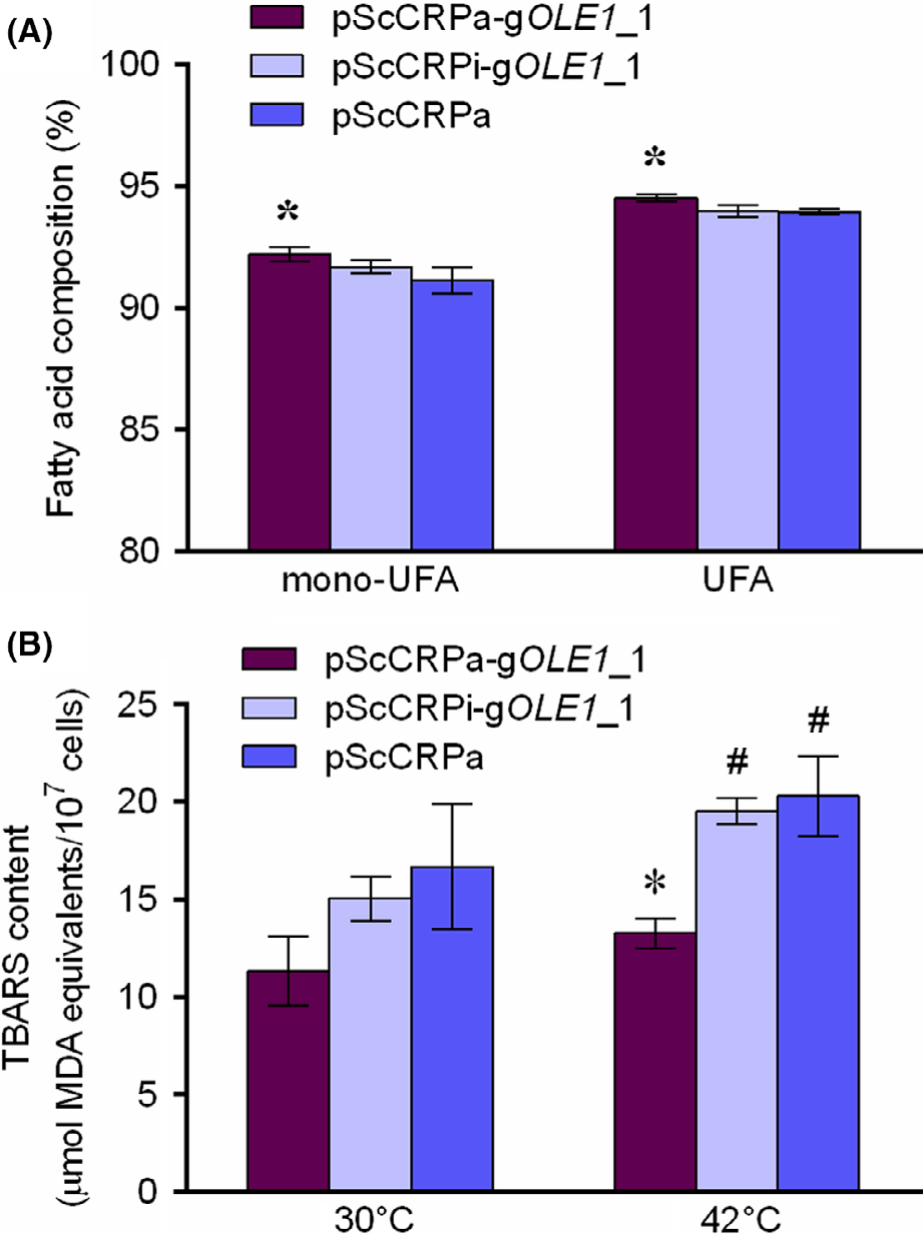
Effects of *OLE1* on (A) fatty acid composition and (B) lipid peroxidation. Yeast cells cultured at 42°C were collected for fatty acid quantification. Fatty acids were converted to fatty acid methyl esters (FAME) and then measured by gas chromatograph (GC). Lipid peroxidation was examined by measuring the level of thiobarbituric acid reactive substances (TBARS). pScCRPa‐g*OLE* *1*_1, *OLE1* overexpression strain; pScCRPi‐g*OLE* *1*_1, *OLE1* knockdown strain as negative control; pScCRPa, strain harbouring blank plasmid pScCRPa as blank control; mono‐UFA, monounsaturated fatty acid; UFA, unsaturated fatty acid; *, significantly different from the control strain harbouring pScCRPa (*P *<* *0.05); ^#^, significantly different from corresponding strains cultured at 30°C (*P *<* *0.05).

The mechanism of thermotolerance associated with *OLE1* was further investigated. In *S. cerevisiae*,* OLE1* encodes the delta‐9 desaturase Ole1, which catalyses the formation of a double bond between C_9_ and C_10_ of CoA‐activated saturated fatty acids (CoA‐SFAs) palmitoyl‐CoA (16:0) and stearoyl‐CoA (18:0), yielding monounsaturated fatty acids palmitoleic acid (16:1) and oleic acid (18:1; Stukey *et al*., 1990). Ole1 is crucial for biosynthesis of unsaturated fatty acids (UFAs) because it is the only known delta‐9 desaturase in *S. cerevisiae,* and delta‐9 desaturase reaction accounts for all *de novo* biosynthesis of unsaturated fatty acids (UFAs; Stukey *et al*., 1990). It was reported that the overexpression of *OLE1* in *S. cerevisiae* enhances ethanol fermentation (Kajiwara *et al*., 2000a,b), ethanol tolerance (Kajiwara *et al*., 2000a,b; Dong *et al*., 2015), cadmium resistance (Fang *et al*., 2017) and tolerance to various stresses (Nasution *et al*., 2017). The mechanisms of these phenotype improvements are usually attributed to the role of *OLE1* in increasing fatty acid unsaturation and enhancing cytoplasmic membrane stability because *S. cerevisiae* maintains membrane fluidity by generating CoA‐activated UFAs as lipid building blocks using Ole1 (Ballweg and Ernst, 2017). Therefore, we analysed the fatty acid composition of different strains in this study (Fig. 4A and Table S3). As shown in Fig. 4A, the strain overexpressing *OLE1* exhibited a slight increase in the percentage of not only monounsaturated fatty acids (mono‐UFAs) but also UFAs compared with those of the blank control strain (*P *<* *0.05), which is consistent with the findings in previous studies (Fang *et al*., 2017; Nasution *et al*., 2017). However, the knockdown of *OLE1* had no significant effect on fatty acid composition. At high temperatures, the generation of reactive oxygen species (ROS) is enhanced significantly as a result of heat stress (Morano *et al*., 2012), leading to lipid peroxidation that triggers cytoplasmic membrane damage (Tsaluchidu and Puri, 2008; Morano *et al*., 2012). It is thought that UFAs may play an antioxidant role (Fang *et al*., 2017). In a recent study, Fang *et al*. (2017) found that the regulation of *OLE1* in the synthesis of UFAs can help yeast cells counter the lipid peroxidation and cytoplasmic membrane damage caused by cadmium. Thus, we assume that *OLE1* also participates in reducing lipid peroxidation at high temperatures. To gain deeper insight into the mechanism of thermotolerance associated with *OLE1*, the effect of *OLE1* on lipid peroxidation was examined by measuring the level of thiobarbituric acid reactive substances (TBARS), a commonly used indicator of lipid peroxidation (Fig. 4B, Fig. S3 and Tables S4–S5). As shown in Fig. 4B, intracellular TBARS content significantly increased when the temperature switched from 30 to 42°C, and intracellular TBARS content in the strain overexpressing *OLE1* was significantly lower than those in the *OLE1* knockdown strain and the blank control strain. These findings demonstrate that the overexpression of *OLE1* can increase fatty acid unsaturation and thereby helped reduce the level of lipid peroxidation induced by heat stress, rendering yeast thermotolerant. As shown in Fig. 4A, the impact of *OLE1* overexpression on fatty acid composition was relatively low compared to those in previous studies (Kajiwara *et al*., 2000a,b; Fang *et al*., 2017; Nasution *et al*., 2017). This was probably due to the relatively low fold change of *OLE1* overexpression in this study. Although 1.65‐fold overexpression of *OLE1* could lead to phenotype change, some of the other 51 candidate genes might not be overexpressed to a high enough degree to observe a phenotype change. As it is difficult to predict which guide sequence would be the most effective in any given promoter, future studies should consider inclusion of multiple guides for each promoter.

## Conclusion and future perspectives

In conclusion, we developed a focused CRISPR/Cas‐based gene activation library in *S. cerevisiae* and used it to screen functional genes that participate in regulating yeast thermotolerance. After high‐temperature screening of the library, we identified a crucial role of the delta‐9 desaturase gene *OLE1* in yeast thermotolerance: the overexpression of *OLE1* increased fatty acid unsaturation, and thereby helped counter lipid peroxidation caused by heat stress, rendering yeast thermotolerant.

In recent years, great efforts have been made to develop thermotolerant yeasts by mutation, genetic engineering, metabolic engineering and physiological adaptation (Choudhary *et al*., 2016). However, it is often difficult to improve phenotypes like thermotolerance using these conventional approaches due to lack of knowledge of their genetic basis or experimental limitations in vector construction, transformation efficiencies and screening capacity (Alper and Stephanopoulos, 2007). This study described the application of CRISPR/Cas‐based gene activation screening with an example of thermotolerant yeast screening, demonstrating that this method can be used for phenotype improvement and identification of functional genes in *S. cerevisiae*. It is thought that large‐scale biofuel production usually requires strains with the following abilities besides thermotolerance: (i) the ability to achieve high cell mass growth and biofuel production rates in biomass‐derived hydrolysates; (ii) the ability to utilize a broad range of pentose and hexose sugars; and (iii) the ability to tolerate low pH (McMillan and Beckham, 2017). In the future, CRISPR/Cas‐based screening can be applied to improve the above abilities of industrial strains. In addition, thermotolerant yeasts such as *K. marxianus* (Limtong *et al*., 2007; Li *et al*., 2018) and *Pichia kudriavzevii* (Yuan *et al*., 2017) are thought to be more suitable for large‐scale biofuel production compared with *S. cerevisiae*. Therefore, the application of CRISPR/Cas‐based screening towards phenotype improvement and identification of functional genes in these yeasts needs to be addressed in the future.

## Experimental procedures

### Strains and plasmids

The strains and plasmids used in this study are listed in Table 1. *Escherichia coli* TOP10 (Tiangen Biotech Co. Ltd., Beijing, China) was used as a host for DNA cloning and plasmid propagation. A wild‐type diploid *Saccharomyces cerevisiae* strain TSH3 (Li *et al*., 2017; Fu *et al*., 2018) was used as the host strain in this study. *E. coli* was grown in LB medium (1% tryptone, 0.5% yeast extract, 1% NaCl). *S. cerevisiae* was grown in YPD medium (1% yeast extract, 2% peptone and 2% glucose).

**Table 1:** Strains and plasmids used in this study

Strains/plasmids	Genotype or description	Source
Strains		
TOP10	*E. coli* TOP10 strain used for molecular cloning	Tiangen Biotech Co. Ltd.
TSH3	Wild‐type *S. cerevisiae* TSH3 strain	Lab collection
TSH3/pScCRPa	*S. cerevisiae* TSH3 harbouring pScCRPa, used as blank control	This study
TSH3/pScCRPa‐g*OLE1*_1	*S. cerevisiae* TSH3 harbouring pScCRPa‐g*OLE1*_1, used to overexpress *OLE1*	This study
TSH3/pScCRPi‐g*OLE1*_1	*S. cerevisiae* TSH3 harbouring pScCRPi‐g*OLE1*_1, used to knockdown *OLE1*	This study
Plasmids		
pTPGI_dCas9_VP64	Yeast CEN/ARS vector that contains dCas9 fused to NLS and VP64 controlled by *TPGI* promoter; selectable marker: *TRP1*	Gift of Dr. Timothy Lu
pTDH3‐dCas9‐Mxi1	Yeast CEN/ARS vector that contains dCas9 fused to NLS and Mxi1 domain controlled by *TDH3* promoter; selectable marker: *LEU2*	Gift of Drs. Stanley Qi & Jonathan Weissman
pScCRPa	Yeast CEN/ARS vector that contains dCas9 fused to NLS and VP64 controlled by *ADH1* promoter; selectable marker: G418	This study
pScCRPi	Yeast CEN/ARS vector that contains dCas9 fused to NLS and Mxi1 domain controlled by *ADH1* promoter; selectable marker: G418	This study
pScCRPa‐g*OLE1*_1	CRISPRa plasmid that contains the specificity sequence of g*OLE1*_1 and is used to overexpress *OLE1*	This study
pScCRPi‐g*OLE1*_1	CRISPRi plasmid that contains the specificity sequence of g*OLE1*_1 and is used to knockdown *OLE1*	This study

The plasmids pTPGI_dCas9_VP64 and pTDH3‐dCas9‐Mxi1 were gifts from Timothy Lu (Addgene plasmid # 49013;Farzadfard *et al*., 2013) and Stanley Qi & Jonathan Weissman (Addgene plasmid # 46921;Gilbert *et al*., 2013), respectively. The plasmid pScLP2‐GFP was constructed in our previous study (Li *et al*., 2017). DNA fragment A containing a dCas9‐VP64 fusion was amplified from pTPGI_dCas9_VP64 using oligonucleotides dCas9‐VP64‐SF2 and VP64‐SR2 as primers. A double‐strand DNA fragment (named as tADH1‐sgRNA), which contains partial *ADH1* terminator and a reversed *SNR52*‐sgRNA‐*SUP4* cassette, was synthesized by SinoGenoMax Co., Ltd (Beijing, China). A NotI site between *SNR52* promoter and the sgRNA sequence was used for the cloning of target specific sequences. DNA fragment B was amplified from tADH1‐sgRNA using primers pScCRP‐F2 and SNR52‐SR. The linearized vector pScLP2‐L was generated via PCR using oligonucleotides pScLP2‐F2 and pScLP2‐R as primers and pScLP2‐GFP as template. The linearized vector pScLP2‐L, fragments A and B were assembled together with EasyGeno Assembly Cloning kit (TIANGEN Biotech Co. Ltd.) to generate plasmid pScCRPa used for CRISPRa‐based site‐specific transcriptional activation (Fig. S1). The plasmid pScCRPi, which contains a dCas9‐Mxi1 fusion from pTDH3‐dCas9‐Mxi1 and a *SNR52*‐sgRNA‐*SUP4* cassette, was constructed by means of a similar method as mentioned above (Fig. S2). This plasmid was used for site‐specific transcriptional repression. The sequences of all the oligonucleotides are listed in Table S6.

### Construction and screening of the gene activation library

We created a library targeting 52 genes based on the results of our previous study (Li *et al*., 2017). Since the expression of *KmHSF1* and *KmMSN2* in *S. cerevisiae* resulted in 31 and 32 up‐regulated genes, respectively (Fold change > 2, *P*_adj_ < 0.05), we chose the union set of these genes as targets in the present study. The oligonucleotides for each sgRNA‐coding sequence for the library were individually designed using CRISPR‐ERA: a comprehensive designer tool for CRISPR genome editing, (gene) repression, and activation, and synthesized by Sangon Biotech (Shanghai, China). Five sgRNAs were designed for each target gene to reduce the influence of off‐target effect. Paired oligonucleotides were designed according to the specificity sequences and the sequence of the sgRNA expression cassette within plasmid pScCRPa. The plasmid pScCRPa was linearized using restriction nuclease NotI. Paired oligonucleotides were annealed separately to make 260 double‐stranded DNAs (5 per target gene). Then, these 260 double‐stranded DNAs were mixed together and ligated into linearized pScCRPa backbone using EasyGeno Assembly Cloning kit (Tiangen Biotech Co. Ltd.) followed by transformation into *E. coli* TOP10 competent cells to obtain the plasmid library. More than 3000 clones of *E. coli* were collected and mixed for plasmid extraction. The plasmid library was then transformed into *S. cerevisiae* TSH3 to obtain a *S. cerevisiae* library for subsequent screening.

The *S. cerevisiae* library was cultured in 200 ml YPD medium at 42°C. After five successive subcultures, the enriched cell culture was diluted and spread onto YPD plates. The same library cultured at 30°C was set as a control group. Then, five colonies were randomly picked for plasmid extraction using Zymoprep II kit (Zymo Research, Orange, CA, USA), followed by PCR amplification of the sgRNA‐coding region. Then, the sgRNA‐coding regions were sequenced using pScCRP‐F2 as primer.

### Real‐time quantitative reverse transcription PCR

Yeast cells were grown to early exponential phase, and then, the total RNA was extracted using EZNA^®^ Yeast RNA Kit (Omega Bio‐tek, Doraville, CA, USA). First‐strand of cDNA was generated from the total RNA using FastKing RT Kit (With gDNase; Tiangen Biotech Co. Ltd.). Then, the generated cDNA was used as qRT‐PCR templates. The gene *TAF10*, which encodes the Taf10 subunit of the TFIID complex, was selected as the reference gene (Teste *et al*., 2009). The qRT‐PCR‐based relative quantification of a target transcript in comparison with the reference gene transcript was performed using Talent qPCR PreMix (SYBR Green; Tiangen Biotech Co. Ltd. Co. Ltd.) on a Step One Plus Real‐Time PCR System (Applied Biosystems, Foster City, CA, USA). The primers for qRT‐PCR are listed in Table S6.

### Cell growth assay

Cultures of *S. cerevisiae* strains were grown in YPD medium containing 200 mg l^−1^ G418 sulfate. Overnight cultures of *S. cerevisiae* grown at 30°C with shaking at 200 rpm were diluted with YPD medium to reach an initial OD_600_ (optical density at 600 nm) of 0.20. These cell suspensions were aliquoted in quadruplicate into sterile 96‐well plates with 200 μl in each well and incubated at 30 or 42°C in a Tecan Infinite M200 Pro plate reader (Tecan Group Ltd., Männedorf, Switzerland) until stationary phase was reached. The OD_600_ values in each well were automatically recorded at intervals of 60 min. Before each measurement, cell cultures were automatically shaken for 90 s to homogenize the samples. In order to quantitatively investigate the growth curves, the growth curve data were fitted with logistic model (Li *et al*., 2018): *y *= *A*_2_ + (*A*_1_ − *A*_2_)/[1 + (*x*/*x*_0_)^*p*^], where *A*_1_, *A*_2_, *x*_0_ and *p* are parameters of logistic model.

Then, the first‐order derivative functions of OD_600_‐time functions were calculated to study the growth speed variations.

For spotting test, 2 μl cell suspensions of each strain with OD_600_ of 0.20 and serial dilutions of 10^−1^ to 10^−3^ were spotted onto YPD agar medium and then incubated at 30°C for 24 h or at 42°C for 96 h.

### Fatty acid quantification

Cell lysis, extraction of total lipids and conversion to fatty acid methyl esters (FAMEs) were based on the protocol of a previous study (Browse *et al*., 1986). An Agilent 7890A gas chromatograph (GC) equipped with a flame ionization detector (FID) was used for analysis. FAME concentrations were calculated by comparing the peak areas in the samples to the peak areas of the high‐purity standards in known concentration relative to the internal standard, respectively.

### Evaluation of lipid peroxidation

As an index of lipid peroxidation, the levels of thiobarbituric acid reactive substances (TBARS) were measured using QuantiChrom TBARS Assay Kit (BioAssay Systems, Hayward, CA, USA) according to the manufacturer's instructions. The amount of malondialdehyde (MDA) produced was determined at 535 nm using a Tecan Infinite M200 Pro plate reader (Tecan Group Ltd., Männedorf, Switzerland). The levels of lipid peroxides were expressed as moles of MDA equivalent per 10^7^ yeast cells.

### Statistical analyses

All experiments were performed with three replicates. Values were expressed as means ± SD. Student's *t* test was used for statistical analyses, in which *P* values of < 0.05 were considered statistically significant.

## Conflict of interest

None declared.

## Supporting information


**Table S1.** sgRNA information in this study.

**Fig. S1.** Map of pScCRPa for CRISPRa‐based site‐specific transcriptional activation.
**Fig. S2.** Map of pScCRPi for CRISPRi‐based site‐specific transcriptional repression.
**Fig. S3.** OD_535_‐MDA calibration curve.
**Table S2.** Fitting results of growth curve data by logistic model.
**Table S3.** Fatty acid composition in different strains.
**Table S4.** Measurement of TBARS content.
**Table S5.** TBARS content.
**Table S6.** Primers and double‐strand DNA used in this study.
