# Supplementary material for: CRISPR/Cas‐based screening of a gene activation library in Saccharomyces cerevisiae identifies a crucial role of OLE1 in thermotolerance
Source: Microb Biotechnol. 2018 Nov 5;12(6):1154–63. doi: 10.1111/1751-7915.13333 (PMC6801138; doi:10.1111/1751-7915.13333)
Supplement: Supplementary file 2 — Fig. S1. Map of pScCRPa for CRISPRa‐based site‐specific transcriptional activation. Fig. S2. Map of pScCRPi for CRISPRi‐based site‐specific transcriptional repression. Fig. S3. OD535‐MDA calibration curve. Table S2. Fitting results of growth curve data by logistic model. Table S3. Fatty acid composition in different strains. Table S4. Measurement of TBARS content. Table S5. TBARS content. Table S6. Primers and double‐strand DNA used in this study. [file MBT2-12-1154-s002.docx]

**Supplementary File 2**

**Article title:** CRISPR/Cas-based screening of a gene activation library in *Saccharomyces cerevisiae* identifies a crucial role of *OLE1* in thermotolerance

**Author names:** Pengsong Li^*^, Xiaofen Fu, Lei Zhang and Shizhong Li^*^

**Affiliation:** MOST-USDA Joint Research Center for Biofuels, Beijing Engineering Research Center for Biofuels, Institute of New Energy Technology, Tsinghua University, Beijing 100084, China

**^*^For correspondence:**

[szli@mail.tsinghua.edu.cn](mailto:szli@mail.tsinghua.edu.cn) (SL);

[lipengsong@mail.tsinghua.edu.cn](mailto:lipengsong@mail.tsinghua.edu.cn) (PL)

**DNA manipulation**


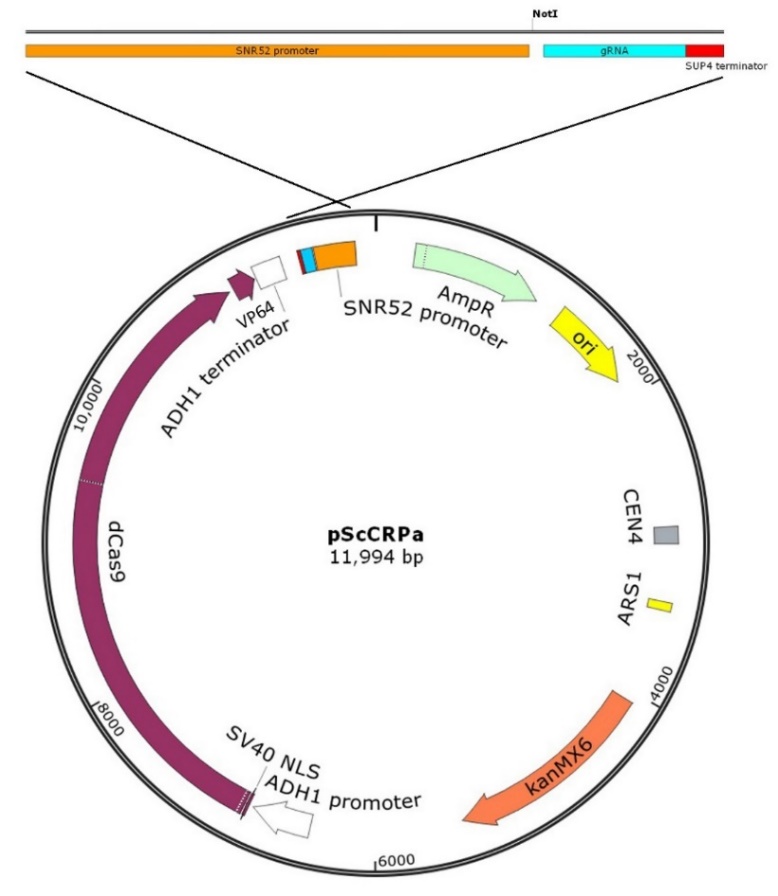


**Fig. S1.** Map of pScCRPa for CRISPRa-based site-specific transcriptional activation.


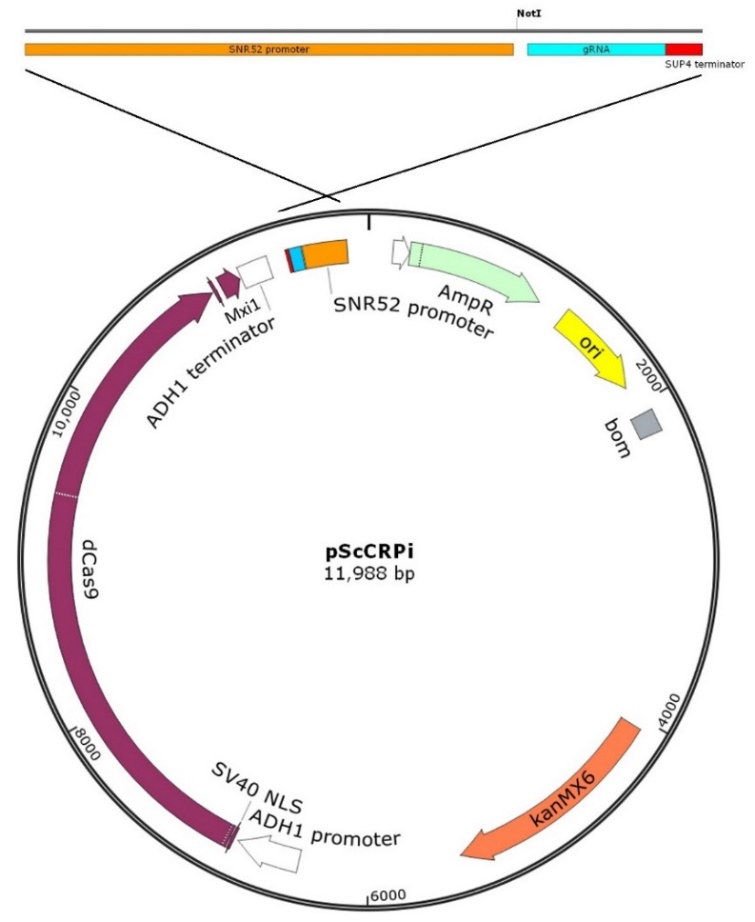


**Fig. S2.** Map of pScCRPi for CRISPRi-based site-specific transcriptional repression.

**Growth curve assay**

**Table S2.** Fitting results of growth curve data by logistic model.

| **Parameters** | | **30 °C** | | | **42 °C** | | |
| --- | --- | --- | --- | --- | --- | --- | --- |
|  |  | **pScCRPa-g*OLE1*** | **pScCRPi-g*OLE1*** | **pScCRPa** | **pScCRPa-g*OLE1*** | **pScCRPi-g*OLE1*** | **pScCRPa** |
| ***A_1_*** | **Value** | 0.27695 | 0.26298 | 0.28406 | 0.26896 | 0.23970 | 0.25058 |
|  | **SD** | 0.00206 | 0.00021 | 0.00324 | 0.00846 | 0.00126 | 0.00170 |
|  |  |  |  |  |  |  |  |
| ***A_2_*** | **Value** | 1.37411 | 1.42318 | 1.34113 | 1.14311 | 1.17706 | 1.20020 |
|  | **SD** | 0.02867 | 0.05655 | 0.01262 | 0.00514 | 0.01057 | 0.01897 |
|  |  |  |  |  |  |  |  |
| ***x_0_*** | **Value** | 5.19602 | 5.83245 | 5.15843 | 11.26976 | 16.14302 | 14.90362 |
|  | **SD** | 0.14751 | 0.41218 | 0.13394 | 0.21556 | 0.13078 | 0.22593 |
|  |  |  |  |  |  |  |  |
| ***p*** | **Value** | 4.77752 | 3.66267 | 6.20924 | 7.74641 | 4.38370 | 4.76555 |
|  | **SD** | 0.26592 | 0.30014 | 0.51220 | 0.68413 | 0.08328 | 0.16521 |
|  |  |  |  |  |  |  |  |
| **R^2^** | **Value** | 0.99804 | 0.99509 | 0.99971 | 0.99778 | 0.99978 | 0.99845 |

Logistic model: $y=A_{2}+\frac{A_{1}-A_{2}}{1+\left( \frac{x}{x_{0}} \right)^{p}}$ , where *A_1_*, *A_2_*, *x_0_* and *p* are parameters of logistic function.

**Fatty acid quantification**

**Table S3.** Fatty acid composition in different strains.

| **Fatty acid (mmol/100g DW)** | **pScCRPa-g*OLE1*** | | | **pScCRPi-g*OLE1*** | | | **pScCRPa** | | |
| --- | --- | --- | --- | --- | --- | --- | --- | --- | --- |
| **C16:0** | 0 | 0 | 0 | 0 | 0 | 0 | 0 | 0 | 0 |
| **C16:1** | 6.744 | 7.238 | 8.814 | 7.451 | 8.677 | 8.770 | 8.414 | 7.681 | 8.886 |
| **C18:0** | 0.821 | 0.872 | 1.062 | 0.961 | 1.161 | 1.043 | 1.070 | 0.999678 | 1.205 |
| **C18:1** | 7.060 | 7.735 | 8.390 | 8.136 | 8.784 | 7.414 | 7.945 | 8.298 | 9.188 |
| **C18:2n6c** | 0 | 0 | 0 | 0.0285 | 0 | 0 | 0 | 0.0317 | 0 |
| **C20:0** | 0 | 0 | 0 | 0 | 0 | 0 | 0 | 0 | 0 |
| **C18:3n6** | 0 | 0 | 0 | 0 | 0 | 0 | 0 | 0 | 0.0126 |
| **C20:1n9** | 0 | 0 | 0 | 0 | 0 | 0 | 0 | 0 | 0 |
| **C18:3n3** | 0.0316 | 0 | 0 | 0 | 0 | 0 | 0 | 0 | 0.0241 |
| **C20:2** | 0 | 0.0182 | 0.0340 | 0 | 0 | 0 | 0 | 0 | 0.0162 |
| **C22:0** | 0 | 0 | 0 | 0 | 0 | 0 | 0 | 0 | 0 |
| **C20:3n6** | 0 | 0 | 0 | 0 | 0 | 0 | 0 | 0 | 0.0131 |
| **C22:1n9** | 0 | 0 | 0 | 0 | 0 | 0 | 0 | 0 | 0 |
| **C20:3n3** | 0 | 0 | 0 | 0 | 0 | 0 | 0 | 0 | 0 |
| **C20:4n6** | 0.00934 | 0 | 0 | 0 | 0 | 0 | 0 | 0 | 0.171 |
| **C22:2n6** | 0 | 0 | 0 | 0 | 0 | 0 | 0 | 0 | 0 |
| **C20:5n3** | 0.0288 | 0.0271 | 0.0324 | 0.0281 | 0.0354 | 0.0393 | 0.0410 | 0.0294 | 0.037 |
| **C24:0** | 0 | 0 | 0 | 0.0350 | 0.0366 | 0 | 0.0339 | 0.0393 | 0 |
| **C24:1n9** | 0.0518 | 0.0464 | 0.0682 | 0 | 0 | 0.0791 | 0 | 0 | 0.0764 |
| **C22:6n3** | 0.306 | 0.287 | 0.368 | 0.301 | 0.371 | 0.426 | 0.451 | 0.349 | 0.402 |
| **mono-UFA percentage** | 92.05% | 92.58% | 92.03% | 92.01% | 91.58% | 91.51% | 91.11% | 91.69% | 90.61% |
| **UFA percentage** | 94.55% | 94.63% | 94.34% | 94.12% | 93.72% | 94.13% | 93.85% | 94.04% | 93.98% |

**Evaluation of lipid peroxidation**

**Table S4.** Measurement of TBARS content.

|  | **Sample/Standard** | **OD_535_** | | |
| --- | --- | --- | --- | --- |
| **Standard 1 (0μM MDA)** | | 0.149 | 0.146 | 0.144 |
| **Standard 2 (9μM MDA)** | | 0.224 | 0.217 | 0.219 |
| **Standard 3 (18μM MDA)** | | 0.301 | 0.303 | 0.305 |
| **TStandard 4 (30μM MDA)** | | 0.412 | 0.429 | 0.305 |
| **30** °C | **pScCRPa-g*OLE1*** | 0.169 | 0.166 | 0.163 |
|  | **pScCRPi-g*OLE1*** | 0.172 | 0.175 | 0.176 |
|  | **pScCRPa** | 0.170 | 0.182 | 0.179 |
| **42** °C | **pScCRPa-g*OLE1*** | 0.171 | 0.169 | 0.172 |
|  | **pScCRPi-g*OLE1*** | 0.184 | 0.185 | 0.182 |
|  | **pScCRPa** | 0.179 | 0.186 | 0.186 |


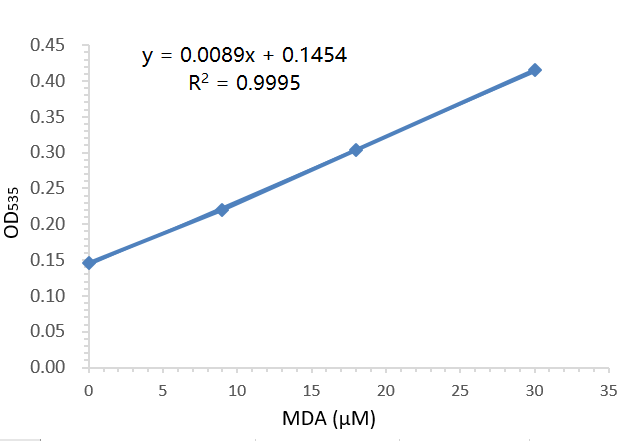


**Fig. S3.** OD_535_-MDA calibration curve

**Table S5.** TBARS content.

|  |  | **TBARS (μmol MDA equivalents/10^7^ cells)** | | | **Mean** | **SD** |
| --- | --- | --- | --- | --- | --- | --- |
| **30** °C | **pScCRPa-****g*OLE1*** | 13.196 | 11.161 | 9.677 | 11.345 | 1.767 |
|  | **pScCRPi-g*OLE1*** | 13.786 | 15.463 | 15.882 | 15.044 | 1.109 |
|  | **pScCRPa** | 13.061 | 19.246 | 17.700 | 16.669 | 3.218 |
| **42** °C | **pScCRPa-g*OLE1*** | 13.488 | 12.422 | 13.914 | 13.275 | 0.769 |
|  | **pScCRPi-g*OLE1*** | 19.549 | 20.163 | 18.833 | 19.515 | 0.666 |
|  | **pScCRPa** | 17.913 | 21.538 | 21.431 | 20.294 | 2.063 |

**Experimental procedures**

**Table S6.** Primers and double-strand DNA used in this study.

| **Name** | **Sequence** |
| --- | --- |
| dCas9-VP64-SF2 | 5’-GGTACCCGGGCCGCCACCATGTCTAGAGCCCCAAAGAAGAAGAGAAAAGTTAGACC-3’ |
| VP64-SR2 | 5’-CTCGAGTTCCTAGGAAAGCATGTCTAAGTCGAAATCGTCC-3’ |
| pScCRP-F2 | 5’-ATGCTTTCCTAGGAACTCGAGGCGAATTTCTTATGATTTATG-3’ |
| SNR52-SR | 5’-GTTAGCATATCTACAATTGGGTTCTTTGAAAAGATAATGTATGATTATGCTTTCACTC-3’ |
| pScLP2-F2 | 5’-ACCCAATTGTAGATATGCTAACTCCAGC-3’ |
| pScLP2-R | 5’-CATGGTGGCGGCCCGGGTACCAGTTG-3’ |
| TAF10-QF | 5’-CACCTTTTCCATCGGTTGCG-3’ |
| TAF10-QR | 5’-CCCATCATCCACTACAGCCTC-3’ |
| OLE1-QF | 5’-TGCCATTGTTACTTTCGGTGA-3’ |
| OLE1-QR | 5’-CCTTAGTTGGGTCGTATTGGT-3’ |
| GPH1-QF | 5’-GACTGGAACAAAACTCAGCAG-3’ |
| GPH1-QR | 5’-CCAAAGCCCTACCCATCAAA-3’ |
| FAS2-QF | 5’-AAGTCTCTAACGGTGGCGTC-3’ |
| FAS2-QR | 5’-ACTCTATTTCTTGCGGGGTGA-3’ |
| HXT6-QF | 5’-TGAAGAGCACGAACCTGTCG-3’ |
| HXT6-QR | 5’-AAACGAAACCACCAAAGGCG-3’ |
| PGI1-QF | 5’-CTGTCTGGTCGGCTATTGGT-3’ |
| PGI1-QR | 5’-GGGGTTTGGGTGAAGTGGTT-3’ |
| ANB1-QF | 5’-GACGGTGAAACCAAAGACGA-3’ |
| ANB1-QR | 5’-TGGCAGAAATGATAGTGACCA-3’ |
| tADH1-gRNA | CCTAGGAACTCGAGGCGAATTTCTTATGATTTATGATTTTTATTATTAAATAAGTTATAAAAAAAATAAGTGTATACAAATTTTAAAGTGACTCTTAGGTTTTAAAACGAAAATTCTTATTCTTGAGTAACTCTTTCCTGTAGGTCAGGTTGCTTTCTCAGGTATAGCATGAGGTCGCTCTTATTGACCACACCTCTACCGGCATGCCGAGCAAATGCCTGCAAATCGCTCCCCATTTCTCTAGAGCGGCCGTGGTATCGTTTAGATTGGCAATTACAGTGTCTTAGCTCACATGCTTATAACTAATTACATGACTCGAAGACATAAAAAACAAAAAAAGCACCGACTCGGTGCCACTTTTTCAAGTTGATAACGGACTAGCCTTATTTTAACTTGCTATTTCTAGCTCTAAAACGCGGCCGCGATCATTTATCTTTCACTGCGGAGAAGTTTCGAACGCCGAAACATGCGCACCAACTTTCACTTCTACAGCGTTTGACCAAAATCTTTTGAACAGAACATTGTAGGGTGTGAAAAAATGCGCACCTTTACCGCTAGCCCAAGAGGGCACTACAAAATCTAGAGTTGTACTTCAAACGTACATGTAATCACCTTGTATATACTCGAAAGAAAACATCAAGTTTCTGTATAAATATGAGTGAAAGCATAATCATACATTATCTTTTCAAAGAACCCAATTGTAGATATGCTAAC |
